# *Salmonella* Neck Abscess as an Opportunistic Infection in Diabetes Mellitus

**DOI:** 10.1155/2013/708419

**Published:** 2013-11-05

**Authors:** Mina Pastagia, Stephen G. Jenkins

**Affiliations:** ^1^Division of Infectious Diseases, Department of Internal Medicine, Mount Sinai School of Medicine, New York, NY 10029, USA; ^2^Department of Microbiology, Weill Cornell School of Medicine, New York, NY 10065, USA

## Abstract

*Salmonella* neck infections represent an uncommon cause of focal salmonellosis. While the incidence of nontyphoid salmonellosis is estimated at over 2 million cases annually, extraintestinal manifestations account for less than 1% of cases. This paper describes two patients with *Salmonella* neck abscesses as the initial presentation of diabetes mellitus. The first patient was diagnosed as having *Salmonella enterica* serotype Enteritidis sternocleidomastoid pyomyositis and the second patient *Salmonella enterica* serotype Typhimurium parapharyngeal abscess. Both patients had elevated hemoglobin A1c levels and had not been previously diagnosed with diabetes mellitus. *Salmonella* spp. should be on the differential as a causative pathogen in patients presenting with neck abscesses and poorly controlled glucose levels. Diabetes may be a risk factor for salmonellosis due to decreased gastric acidity and prolonged gastric transit time. Prompt incision and drainage accompanied by antibiotics remains the treatment of choice for infected neck abscesses.

## 1. Introduction

*Salmonella *infections are typically classified into four categories: gastroenteritis, enteric fever, focal disease, and chronic carrier state. The infection may be localized to the gastrointestinal tract or may disseminate via the blood or lymphatic system. Focal salmonellosis is thought to be secondary to a brief episode of bacteremia after infection from the GI tract. Patients with significant underlying conditions are at increased risk for the development of focal infection. This has been observed in patients with HIV, diabetes, and malignancy [1].

Pyomyositis, an intramuscular abscess of skeletal muscle, was first described in the tropics. The pathogenesis most likely involves transient bacteremia in addition to prior or concurrent muscle damage. *Staphylococcus aureus* is the most common microorganism responsible for both tropical and temperate cases [2]. The median age, rate of underlying conditions, and mortality in patients with *Salmonella *muscle infections are significantly higher than those patients presenting with pyomyositis involving other microorganisms [3]. Here we present a case of sternocleidomastoid pyomyositis caused by *Salmonella enterica *serotype Enteritidis, as well as a parapharyngeal space abscess caused by *Salmonella enterica *serotype Typhimurium in two patients newly diagnosed with uncontrolled diabetes mellitus.

## 2. Case 1

A 46-year-old woman with a past medical history significant for hypertension presented to the ED with an eight-week history of increasing pain and swelling of the right neck. Over the past 3 weeks the patient had developed odynophagia to solid foods and had intermittent fevers and chills. She denied any recent neck trauma but admitted to a nearly 30 lb. weight loss over the past two months. She denied recent dental pain and dental work. She did not have any recent abdominal pain or diarrhea. There was no history of raw milk consumption or undercooked meat exposure.

**Figure 1:**
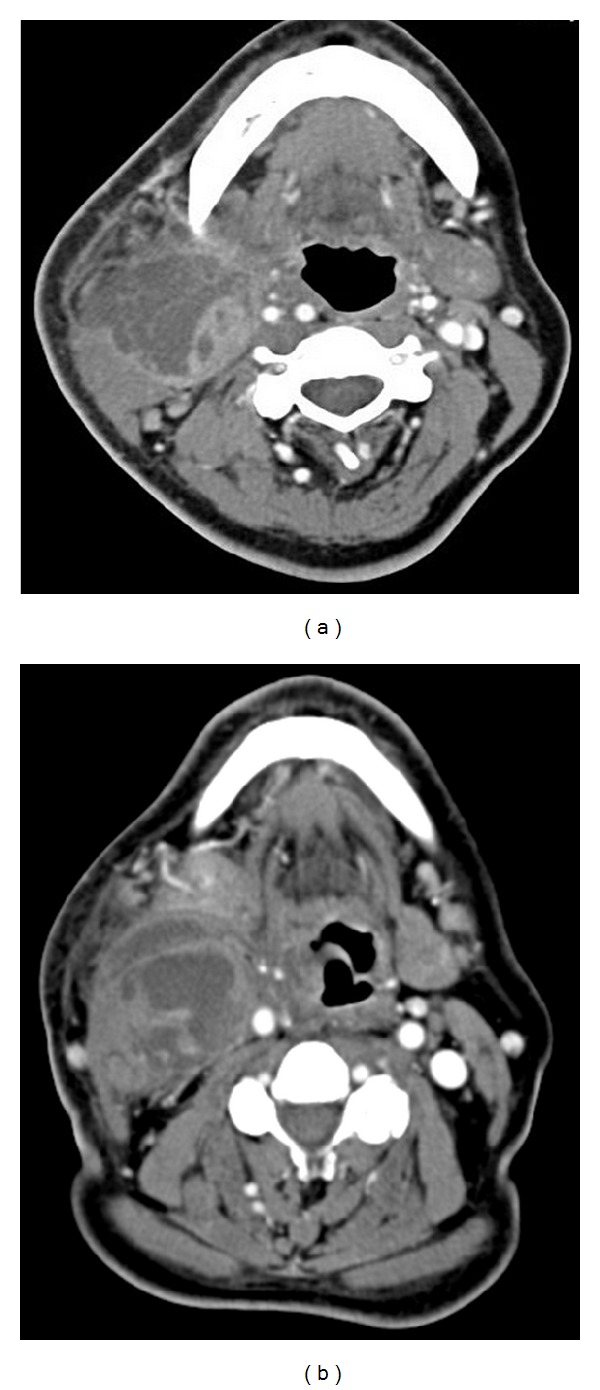
Neck mass imaging. (a) Computed tomography appearance in Case 1 of the multiloculated abscess involving the right sternocleidomastoid muscle. (b) Computed tomography appearance in Case 2 of the multiloculated abscess involving the right parapharyngeal space. Both patients required surgical drainage for pathogen identification and treatment.

The patient was born in Puerto Rico and immigrated to New York one month before her symptoms. She denied history of tuberculosis contacts or intravenous drug use and had never been tested for HIV. The patient had one kitten and several ducks as pets. On presentation the patient was tachycardic and febrile (39°C). Physical exam revealed a tender, erythematous, nonfluctuant 5 × 6 cm mass in the right neck extending from the angle of the mandible to the hyoid bone. No other lymphadenopathy was identified. Oral cavity exam revealed good dentition. Flexible fiberoptic nasolaryngoscopy was unremarkable. Initial laboratory studies revealed a white blood cell count of 12.5 × 10^9^/dL, with 89% polymorphonuclear leukocytes, and serum glucose of 350 mg/100 mL. Chest X-ray demonstrated clear lung fields, with a right sided neck mass. A computed tomgraphy (CT) scan was performed demonstrating a 4.6 × 4.2 × 6 cm multiloculated mass in the right neck involving the sternocleidomastoid muscle (Figure 1(a)). 

**Figure 2:**
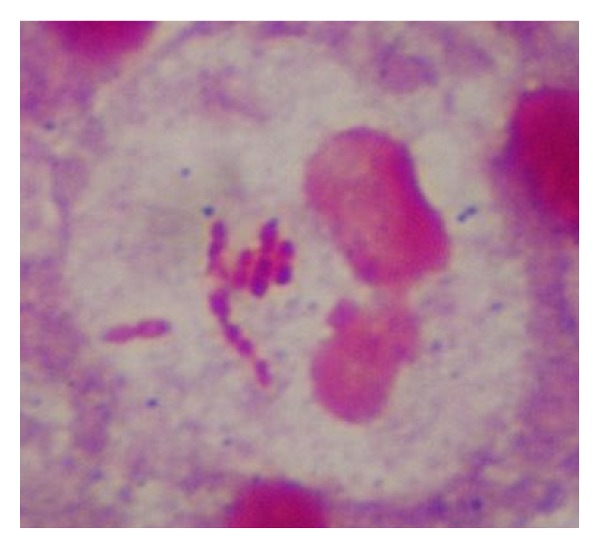
Case 1 Gram stain. Gram-negative bacilli seen intracellularly (100x magnification).

**Figure 3:**
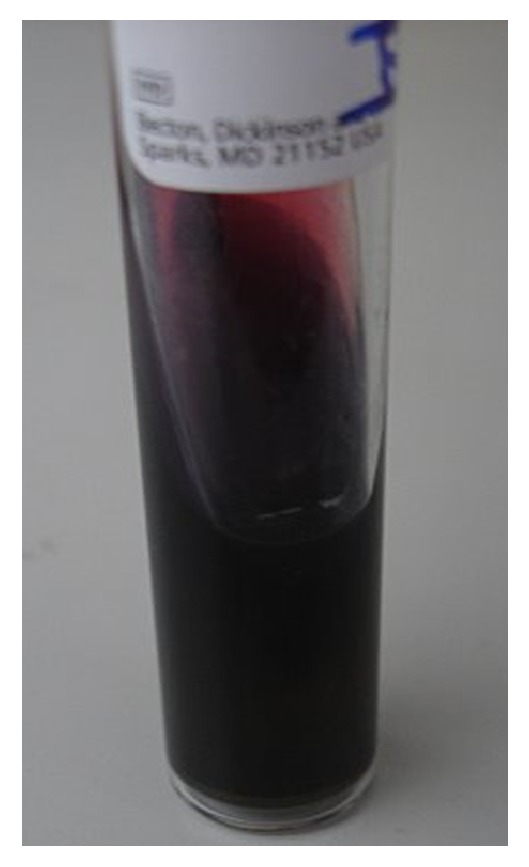
Triple sugar iron (TSI) slant. Hydrogen sulfide production is typically seen with *Salmonella* spp. and serves as a diagnostic aid.

The patient was immediately taken to the operating room for incision and drainage of the neck mass. Intraoperatively 20 cc of purulent discharge was evacuated and a Penrose drain was placed. The patient was given 3 grams IV ampicillin-sulbactam every six hours and insulin was used for blood glucose control. A Mantoux purified protein derivative (ppd) was placed which was subsequently negative. The patient had an uneventful postoperative course with normalization of her white blood count on postoperative day number 2. The Gram stain of the abscess culture revealed intracellular Gram-negative bacilli (Figure 2). The TSI slant result was alkaline/acid with production of hydrogen sulfide gas (Figure 3). The patient was switched to oral ciprofloxacin 500 mg twice daily on postoperative day 3 when cultures grew back *Salmonella enterica *serotype Enteritidis sensitive to ceftriaxone, ampicillin, ciprofloxacin, trimethoprim-sulfamethoxazole, and ertapenem. No other pathogens were recovered. Ciprofloxacin susceptibility was verified via E-test. Blood cultures as well as stool cultures were negative. A right upper quadrant ultrasound revealed no evidence of cholecystitis. A human immunodeficiency virus (HIV) test was negative. Hemoglobin A1c was elevated at 12.6%. The patient was discharged home on postoperative day number five with resolution of symptoms to complete a fourteen-day course of ciprofloxacin with appropriate blood glucose control.

## 3. Case 2

A 55-year-old man with no known past medical history presented to the hospital with a four-week history of swelling and pain in the right side of the neck. The patient experienced dysphagia to solids as well as subjective fever accompanied by chills. He denied any trauma to the neck but did have 15 lb. weight loss. He did not have any recent dental pain or dental work. He had not experienced abdominal pain or diarrhea over the preceding month but did admit to polydipsia and polyuria. There was no history of raw milk consumption or undercooked meat exposure. The patient was born in Mexico and had been living in New York for three years, without any other travel history. He denied history of tuberculosis exposure or intravenous drug use and had never been tested for HIV. He had no pets in New York or in Mexico. On presentation the patient was mildly tachycardic and febrile (38.5°C). Physical exam revealed a tender, erythematous, nonfluctuant 6 × 7 cm mass in the right neck extending from the mandible of the jaw. No other lymphadenopathy was identified. Oral cavity exam revealed good dentition. Initial laboratory studies were remarkable for a white blood cell count of 8.6 × 10/dL, with 79% polymorphonuclear leukocytes and serum glucose of 180 mg/100 mL. Chest X-ray revealed clear lung fields. A computed tomography (CT) scan of the neck demonstrated a 4 × 7 cm multiloculated mass in the right neck involving the parapharyngeal space with extension around the right carotid artery (Figure 1(b)).

The patient was started on 3 grams of ampicillin-sulbactam intravenously every six hours. A scant amount of pus expressed superficially was sent for culture. Two days later the patient was taken to the operating room for incision and drainage. Intraoperatively 30 cc of purulent discharge was evacuated and a Penrose drain was placed. Insulin was used for blood glucose control. Two sets of blood cultures were negative for infection. The Gram stain of the abscess culture revealed Gram-negative bacilli with the TSI slant alkaline/acid. The culture yielded *Salmonella enterica *serotype Typhimurium, sensitive to ceftriaxone, ampicillin, ciprofloxacin, trimethoprim-sulfamethoxazole, and ertapenem. No other pathogens were recovered. Ciprofloxacin susceptibility was confirmed via E-test. Stool cultures and an HIV test were negative. A right upper quadrant ultrasound revealed no evidence of cholecystitis. The patient's hemoglobin A1c was 9.6%. The patient was discharged on postoperative day number four with resolution of symptoms to complete a fourteen-day course of oral ciprofloxacin 500 mg twice daily along with metformin for glucose control.

## 4. Discussion

While the incidence of nontyphoid salmonellosis is estimated at over 2 million cases annually, extraintestinal manifestations account in less than 1% of these cases [4]. In nonserotype Typhi strains, transmission usually occurs via animal reservoirs such as chickens, eggs, or via pets such as turtles, reptiles. *Salmonella* infection acquired through the gastrointestinal tract has two possible routes of systemic spread. *Salmonella* can invade the bloodstream causing bacteremia and then seed to distant sites or it can enter the lymphatics of the gastrointestinal tract or the tissue of the tonsil directly through the oropharynx and spread via the lymphatics. The bacterium itself can live for unknown periods of time in macrophages, resulting in a continuous carrier state. Distal infection sites can present long after an acute gastrointestinal illness. Remote abscesses are the result of hematogenous or lymphatic dissemination of primary gastrointestinal tract infections [1].

Salmonellae species are Gram-negative motile bacilli that are facultative anaerobic and nonlactose fermenting. They produce acid on glucose fermentation, reduce nitrates, and do not produce cytochrome oxidase, similar to other Enterobacteriaceae. Approximately 1% of organisms are able to ferment lactose and may not be detected if only MacConkey agar or other semiselective media are used [1]. Both patients' isolates were detected on MacConkey agar. A TSI slant was also used to reveal production of hydrogen sulfide by the organisms. The Gram stain in Figure 2 revealed intracellular Gram-negative bacilli. Diabetes, chronic liver disease, malignancy, aplastic anemia, sickle cell anemia, chronic steroid use, dialysis, and chemotherapy treatment are known risk factors [5].

Pyomyositis, an intramuscular abscess of skeletal muscle, is often described in three stages. The first (invasive) stage results in bacterial seeding into the muscle without abscess formation. The second (suppurative) stage is characterized by abscess formation. Ninety percent of cases are recognized at this stage. The third stage is characterized by septicemia, metastatic disease, and confers a high mortality [6]. Diagnosis must be confirmed by ultrasound, CT scan, or magnetic resonance scanning. Treatment of stage 1 includes antibiotics alone; for stages 2 and 3, incision and drainage in addition to antibiotics is recommended. Prognosis with treatment tends to be quite good [7]. Most cases of bacterial pyomyositis are due to *Staphylococcus aureus*. The most frequent locations include psoas, quadriceps, and buttock muscles. Location in the neck has been reported in 0.4% [1]. It has been postulated that fibronectin binding receptors on muscle cells may be the route for bacterial entry and that prior muscle injury facilitates the development of pyomyositis. Certain immunocompromised states may induce unrecognized muscle injury, leading to increased risk for pyomyositis [8].

Medina et al. have described two cases of *Salmonella enterica, *serotype Enteritidis pyomyositis of the quadriceps and gluteus muscles in HIV patients [9]. A Medline review by Crum of bacterial pyomyositis among HIV and non-HIV infected persons in the United States showed that pyomyositis among HIV-infected persons is more often associated with end-stage disease (CD4 < 50 cells/mm) with an increased risk for recurrence. In the USA, non-HIV patients with pyomyositis usually have underlying medical conditions such as diabetes or malignancy [8].

A Medline review by Collazos et al. of *Salmonella* muscle infections revealed that the median age was significantly higher in patients with *Salmonella *muscle infections than in patients with typical pyomyositis (53.5 versus 30 years of age). The rate of underlying conditions was also significantly higher in *Salmonella* muscle infections. The main immunocompromising conditions included diabetes and HIV infection. The rate of psoas muscle involvement was markedly higher, as was the presence of bacteremia. *Salmonella enterica* serotype Enteritidis was noted to be the most frequent serotype involved. Most importantly, those patients with *Salmonella *muscle infections had a considerably higher mortality rate, involving one-third of patients, as compared to that of pyomyositis involving other microorganisms [3].

Other types of neck abscesses involving *Salmonella* spp. have been described in the literature. A recent report by Luo and Liu demonstrated two cases of neck abscess and necrotizing fasciitis caused by *Salmonella enterica *serotype Enteritidis in diabetic patients [10]. Yamagata et al. [11] and Bahar et al. [5] have described submandibular abscesses caused by *Salmonella enterica *serotype Enteritidis in two diabetic patients with history of dental disease. A large review by Su et al. [12] described nineteen patients with *Salmonella* neck abscesses, with the first reported case in 1935. Fifteen patients were adults, and nine of these had diabetes mellitus. Gudipati and Westblom reported a case of *Salmonella *serotype Typhimurium thyroid abscess in 1991 [13]. Three other *Salmonella *thyroid abscesses have been described, with two of those being caused by *Salmonella enterica* serotype Typhimurium and one being caused by *Salmonella enterica* serotype Panama [14].

It is unclear how the patients described here developed infection with *Salmonella* spp. Neither of the patients was chronic carriers. Both patients were previously undiagnosed diabetics with poor glucose control. In a case-control study by Telzak et al., patients in a New York City hospital who developed infection after exposure to an *S. enteritidis*-contaminated meal were more likely to be diabetics than those who did not develop infection (17/75 versus 7/80, Mantel-Haenszel adjusted odds ratio = 3.1, 95% confidence interval = 1.1, 8.6). Proposed mechanisms for diabetes as a risk factor for infection include decreased gastric acidity and an autonomic neuropathy of the small bowel that reduces intestinal motility and prolongs gastrointestinal transit time [15].

## 5. Conclusions

In immunocompromised patients, *Salmonella *species must remain in the differential for any muscle or neck abscess. These patients must be screened for diabetes mellitus and maintain strict glucose control. Recovery of *Salmonella* confers a higher risk for mortality, and appropriate treatment includes prompt incision and drainage in addition to antibiotics.

Written informed consent was obtained from the patients for publication of this case series and accompanying images.
